# Isolation and Crystal Structure of Marcanine A from *Polyalthia plagioneura*

**DOI:** 10.3390/molecules15096349

**Published:** 2010-09-09

**Authors:** Bingjing Liu, Lin Wang, Guangying Chen, Changri Han, Jing Wang

**Affiliations:** 1 Key Laboratory of Tropical Medicinal Plant Chemistry of Ministry of Education; Hainan Normal University, Hainan 571158, China; 2 College of chemistry and chemical engineering, Hainan Normal University; Hainan 571158, China

**Keywords:** : *Polyalthia plagioneura*, marcanine A, NMR, X-ray diffraction, anticancer activities

## Abstract

Marcanine A was isolated from the stems of *Polyalthia plagioneura* as light yellow crystals. The molecular and crystal structures have been determined by 1D,2D-NMR and X-ray diffraction analysis. It crystallizes in the triclinic system, space group P-1 with a = 5.2140(5)Å, b = 10.1871(11)Å, c = 11.0709(13)Å, α = 110.452(2)º, β = 103.376(2)°, γ = 90.1870(10)°, V = 533.74(10)Å^3^, Z = 2. There are three intermolecular hydrogen bonds in a unit cell. It displays some inhibitory activities towards four kinds of human tumor cells, including BEL-7402, K562, SPCA-1and SGC-7409.

## 1. Introduction

**Figure 1:**
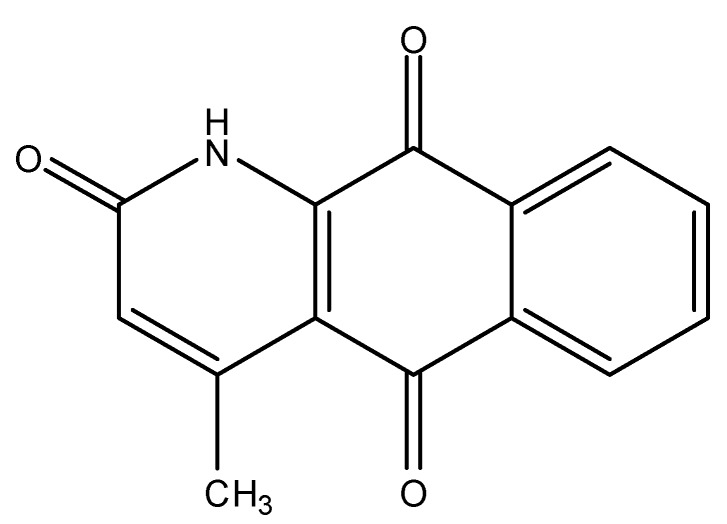
Chemical structure of marcanine A.

*Polyalthia plagioneura* (Annonaceae) is a typical medium-sized tree in P.R. China, occurring mainly in Hainan, Guangdong, Guangxi and Yunnan provinces [1]. In a previous study only two compounds, howiicin A and plagionicin A were isolated from *P. plagioneura* [2,3]. In the present paper, we report marcanine A (Figure 1) isolated from this plant as light yellow crystals and its single-crystal structure determination by X-ray diffraction analysis. Marcanine A was isolated from *Polyalthia* genus for the first time.

It is reported that marcanine A showed several biological activities. Soonthornchareonnon *et al*. [4] reported the cytotoxicity of marcanine A, which displayed IC_50_ values between 80 nM and 2.1 μM against several human tumor cells, including A-549, HT-29, MCF7, RPMI and U251. Ichino *et al*. [5] found marcanine A had *in vitro* antimalarial activity against the drug-resistant K1 strain of *Plasmodium falciparum*.

## 2. Results and Discussion

### 2.1. Crystal structure determination

**Figure 2:**
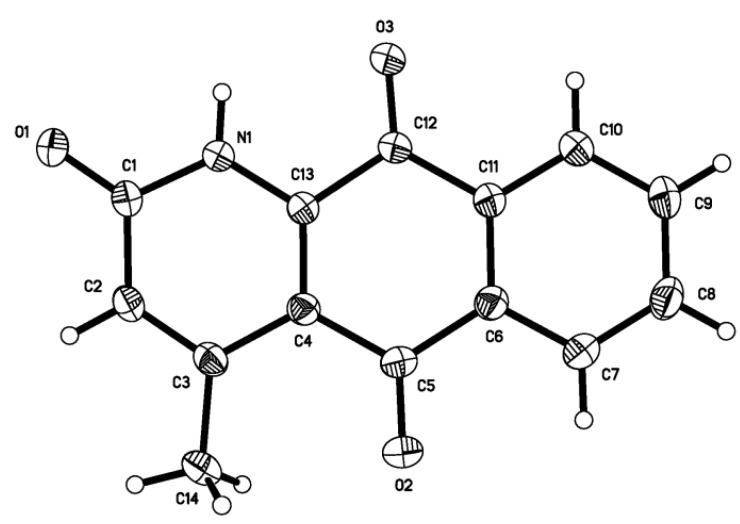
Molecular structure of the title compound.

**Figure 3:**
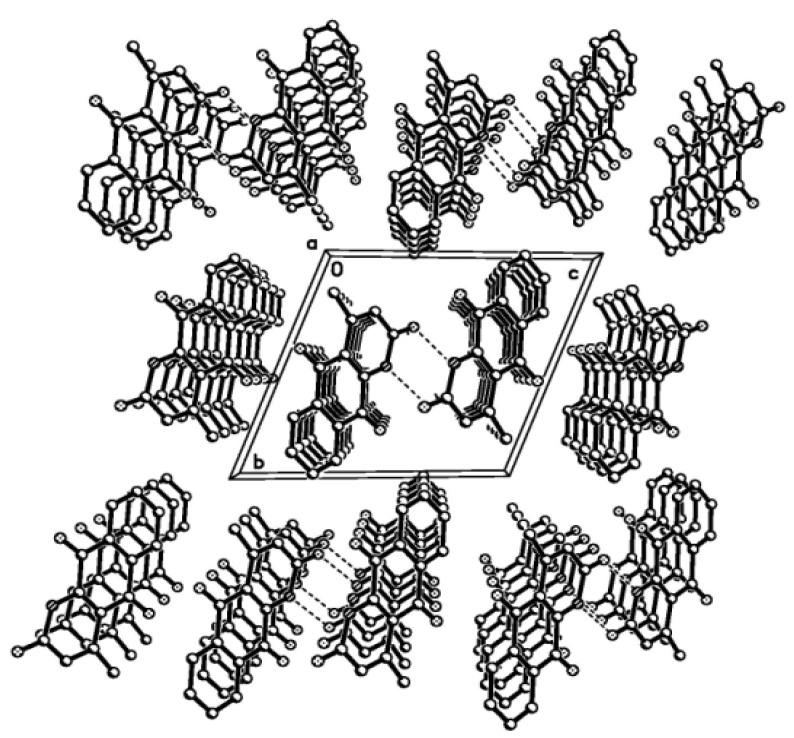
Packing of the molecules in a unit cell.

The molecular structure of the title compound was elucidated on the basis of physicochemical properties and spectral data including IR,1D-NMR and 2D-NMR. And the molecular formula of the crystal is C_14_H_9_NO_3_ (Mr = 239.22). Its molecular structures is shown in Figure 2, and the hydrogen bonding diagram and packing diagram in a unit cell are shown in Figure 3, respectively. A summary of the crystal data and refinements is listed in Table 1. The thermal parameters of non-hydrogen atoms are given in Table 1. The selected bond lengths, bond angles and hydrogen bond lengths and angles are given in Table 2 and Table 3, respectively.

**Table 1:** Crystal Data and Structural Refinements.

Empirical formula	C_14_H_9_NO_3_	Volume (Å^3^)	533.74(10)
Formula weight	Mr =239.22	Absorption coefficient (mm^-1^)	0.106
Z	2	F(000)	248
Dc (Mg/m^3^)	1.489	Crystal size mm)	0.50× 0.42 × 0.41
Color, shape	light yellow, block	θ range for data collection	2.03-25.01
Temperature (K)	293K	Index ranges	*h* = −6→6
Wavelength (Å)	0.71073		*k* = −8→12
Crystal system	Triclinic		*l* = −13→9
Space group	P-1	Reflections collected	2776
Cell dimensions		Independent reflections	1849
a(Å)	5.2140(5)	R_int_	0.0148
b(Å)	10.1871(11)	F^2^	1.032
c(Å)	11.0709(13)	Max. and min. transmission	0.9577 and 0.9488
α(º)	110.452(2)	Data/restraints/parameters	1849/0/164
β(º)	103.376(2)	Final R indices (I > 2σ(I))	R=0.041
γ(º)	90.1870(10)		wR=0.1104

**Table 2:** Selected Bond Lengths (Å) and Bond Angles (°).

Bond	Dist.	Bond	Dist.	Bond	Dist.
N(1)-C(13)	1.352(2)	N(1)-C(1)	1.383(2)	O(3)-C(12)	1.2156(18)
O(1)-C(1)	1.237(2)	O(2)-C(5)	1.214(2)	C(4)-C(13)	1.372(2)
C(2)-C(3)	1.358(2)	C(6)-C(11)	1.398(2)	C(3)-C(4)	1.449(2)
C(1)-C(2)	1.431(3)	C(3)-C(14)	1.499(2)	C(6)-C(7)	1.392(3)
C(4)-C(5)	1.481(2)	C(5)-C(6)	1.493(3)	C(9)-C(10)	1.383(3)
C(7)-C(8)	1.379(3)	C(8)-C(9)	1.379(3)	C(12)-C(13)	1.499(2)
C(10)-C(11)	1.393(2)	C(11)-C(12)	1.476(2)		
**Angle**		(°)	**Angle**		(°)
N(1)-C(1)-C(2)		114.28(15)	O(3)-C(12)-C(11)		123.66(15)
O(1)-C(1)-N(1)		120.79(16)	N(1)-C(13)-C(12)		114.72(14)
C(13)-N(1)-C(1)		123.43(14)	O(1)-C(1)-C(2)		124.93(16)
O(2)-C(5)-C(6)		120.10(16)	C(6)-C(11)-C(12)		119.95(15)
O(3)-C(12)-C(13)		118.93(15)	C(3)-C(2)-C(1)		124.54(16)
C(2)-C(3)-C(14)		118.98(15)	C(2)-C(3)-C(4)		117.64(16)
C(13)-C(4)-C(3)		117.98(15)	C(4)-C(3)-C(14)		123.37(16)
C(3)-C(4)-C(5)		122.70(15)	C(13)-C(4)-C(5)		119.32(15)
C(4)-C(5)-C(6)		118.36(15)	O(2)-C(5)-C(4)		121.53(16)
C(7)-C(6)-C(5)		119.17(16)	C(7)-C(6)-C(11)		119.16(16)
C(8)-C(7)-C(6)		120.08(18)	C(11)-C(6)-C(5)		121.67(15)
C(8)-C(9)-C(10)		119.82(17)	C(9)-C(10)-C(11)		119.97(17)
C(10)-C(11)-C(6)		120.10(16)	C(11)-C(12)-C(13)		117.39(14)
C(9)-C(8)-C(7)		120.87(18)	C(10)-C(11)-C(12)		119.95(15)
N(1)-C(13)-C(4)		122.09(14)	C(4)-C(13)-C(12)		123.15(15)
C(13)-N(1)-C(1)-O(1)		-178.72(16)	C(13)-N(1)-C(1)-C(2)		0.5(3)
O(1)-C(1)-C(2)-C(3)		-179.75(18)	N(1)-C(1)-C(2)-C(3)		1.0(3)
C(1)-C(2)-C(3)-C(4)		-0.8(3)	C(1)-C(2)-C(3)-C(14)		-179.30(18)
C(2)-C(3)-C(4)-C(13)		-1.1(3)	C(14)-C(3)-C(4)-C(13)		177.43(17)
C(2)-C(3)-C(4)-C(5)		179.82(17)	C(14)-C(3)-C(4)-C(5)		-1.7(3)
C(13)-C(4)-C(5)-O(2)		175.98(18)	C(3)-C(4)-C(5)-O(2)		-4.9(3)
C(13)-C(4)-C(5)-C(6)		-4.4(3)	C(3)-C(4)-C(5)-C(6)		174.76(15)
O(2)-C(5)-C(6)-C(7)		2.3(3)	C(4)-C(5)-C(6)-C(7)		-177.41(16)
O(2)-C(5)-C(6)-C(11)		-178.83(18)	C(4)-C(5)-C(6)-C(11)		1.5(3)
C(11)-C(6)-C(7)-C(8)		-0.3(3)	C(5)-C(6)-C(7)-C(8)		178.65(17)
C(6)-C(7)-C(8)-C(9)		0.0(3)	C(7)-C(8)-C(9)-C(10)		0.4(3)
C(8)-C(9)-C(10)-C(11)		-0.5(3)	C(9)-C(10)-C(11)-C(6)		0.2(3)
C(9)-C(10)-C(11)-C(12)		-179.87(17)	C(7)-C(6)-C(11)-C(10)		0.2(3)
C(5)-C(6)-C(11)-C(10)		-178.72(17)	C(7)-C(6)-C(11)-C(12)		-179.71(17)
C(5)-C(6)-C(11)-C(12)		1.4(3)	C(10)-C(11)-C(12)-O(3)		0.0(3)
C(6)-C(11)-C(12)-O(3)		179.91(17)	C(10)-C(11)-C(12)-C(13)		178.61(16)
C(6)-C(11)-C(12)-C(13)		-1.5(3)	C(1)-N(1)-C(13)-C(4)		-2.4(3)
C(1)-N(1)-C(13)-C(12)		175.17(15)	C(3)-C(4)-C(13)-N(1)		2.6(3)
C(5)-C(4)-C(13)-N(1)		-178.24(16)	C(3)-C(4)-C(13)-C(12)		-174.76(15)
C(5)-C(4)-C(13)-C(12)		4.4(3)	O(3)-C(12)-C(13)-N(1)		-0.4(2)
C(11)-C(12)-C(13)-N(1)		-179.02(15)	O(3)-C(12)-C(13)-C(4)		177.18(17)
C(11)-C(12)-C(13)-C(4)		-1.5(3)			

**Table 3:** Hydrogen Bond Lengths (Å) and Bond Angles (°).

D-H···A	D-H	H‑A	D···A	D-H···A
N (1)-H (1)···O(1)^a^	0.860	2.05	2.880	162
C (10)-H (10)···O(3)^b^	0.93	2.45	3.245(2)	143
C (7)-H (7)···O(2)^c^	0.93	2.38	3.279(2)	162

Symmetry code: ^a^*-x,1-y,1-z*; ^b^*1-x,2-y,1-z*; ^c^ *2-x,1-y,-z.*

From Table 2, it can be seen that all of bond angles are larger than 110° and less than 125°. The molecule contains three six-membered rings. In the amide ring (ring A) the bond distances and angles around N1 and O1 are in keeping with the geometric parameters found in intra-amides which are conjugated with a carbon-carbon double bond. All atoms in ring A are nearly coplanar ,C(1), C(2), C(3) C(4) and C(13) are almost in a plane controlled by the three double bonds [C(1)=O(1),C(2)=C(3) and C(4)=C(13)]. In the six-membered ring of the benzoquinone group (ring B), all atoms are strictly coplanar, that is C(4), C(5), C(6) C(11), C(12) and C(13) are also almost in a plane controlled by the four double bonds [C(5)=O(2), C(12)=O(3),C(6)=C(11) and C(4)=C(13)], respectively.

Table 3 gives the hydrogen-bonding geometry. Owing to the peculiar spatial arrangement of the crystal, a few noticeable intermolecular hydrogen bonds are formed. Two molecules are connected by three intermolecular hydrogen bonds in a unit cell. A N-H group at N(1) forms an intermolecular hydrogen bond. The oxygen atom at C(1) has the C=O group as its acceptor. The value suggests that all the six member ring atoms are parallel in packing. Indeed, the stacking interaction also exhibits a planar molecular array (Figure 3). These intermolecular conventional and unconventional interactions link the molecules into an infinite two-dimensional supramolecular network structure and play key roles in stabilizing the crystal packing.

### 2.2. Cytotoxicity

From Table 4, we can learn that the title compound has some inhibitory activity towards four kinds of tumor cells. The IC_50_ were less than 12 μM, which works most effective on SGC-7409 (IC_50_ = 1.53 μM), and relatively weak on K562 (IC_50_ = 11.78 μM).

**Table 4:** Evaluation of the cytotoxic activity (IC_50_/μM) of title compound against human tumor cell lines.

Tumor cell species	Inhibition(%)						IC_50_/μM
**0.1μM**	**1μM**	**5μM**	**10μM**	**50μM**	**100μM**
BEL-7402	-28.55	-30.16	2.42	30.07	100.12	100.46	9.54
K562	-46.03	-0.25	19.93	33.67	92.15	102.25	11.78
SPCA-1	-19.45	8.43	6.20	44.37	99.50	97.61	8.69
SGC-7409	13.58	14.16	42.12	71.87	119.73	122.45	1.53

## 3. Experimental

### 3.1. Plant material

The stems of *P. plagioneura* were collected from BaWangling mountain in Hainan Province, P.R. China in May 2008, and identified as *Polyalthia plagioneura* by vice-professor Qiongxin Zhong from the College of Life Science in Hainan Normal University. A voucher specimen has been preserved in the Key Laboratory of Tropical Medicinal Plant Chemistry of Ministry of Education, Hainan Normal University.

### 3.2. Extraction and separation

Air-dried stems of *P.plagioneura* (20 kg) were ground and percolated (4 × 3 h) with 75% EtOH at 60 ºC, which was suspended in 5 L water and then successively partitioned with chloroform, ethyl acetate and *n*-BuOH, yielding a chloroform extract, an ethyl acetate extract and a *n*-BuOH extract, respectively. The chloroform extract was subjected to a silica gel CC column using petroleum ether as first eluent and then increasing the polarity with EtOAc, to afford 33 fractions. Fraction 6 was further separated by column chromatography with a gradient of petroleum ether-EtOAc to give the title compound.The crude product was recrystallised from chloroform to yield light yellow blocks.

### 3.3. Sructure determination

M.p. 249-251 ºC; IR max (KBr) cm^-1^: 3462, 2128, 1643, 1461, 1398, 1292, 1162, 924, 858, 728; ^1^H-NMR (Bruck AV-400), (400 MHz in CDCl_3_), ppm: 2.71 (3H, d, *J* = 0.8 Hz, C_3_-CH_3_), 6.67 (1H, d, *J* = 0.8 Hz, H-2), 7.83 (1H, dt, *J* =7.6, 1.2 Hz, H-9),7.87 (1H, dt, *J* = 7.6, 1.6Hz, H-8), 8.19 (1H, dd, *J* = 7.6, 0.8 Hz, H-7),8.24 (1H, dd, *J* = 7.6, 1.2 Hz, H-10); ^13^C-NMR (100 MHz in CDCl_3_), ppm 178.0 and 181.4 (CO-12 & CO-5), 160.3 (CO-1), 152.2 (C-13), 139.8(C-3), 135.8 (C-9), 133.7 (C-8), 133.3 (C-11), 130.0 (C-6),127.7 (C-2), 127.5 (C-10), 126.7 (C-7) and 116.1 (C-4), 22.7 (CH_3_) [6,7]. In the HMBC it exhibited correlations of H-2 to C-1; H-7 to C-5 and C-6 and C-8; H-8 to C-10; H-9 to C-7 and C-8; H-10 to C-11 and C-12 and methyl protons to C-2 and C-4. On the basis of the above data, the structure was elucidated as marcanineA (Figure 4).

**Scheme 4:**
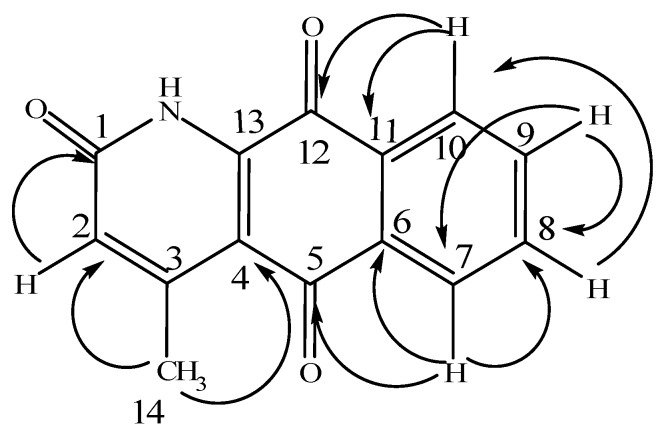
Key HMBC correlations for the title compound.

### 3.4. Crystal structure determination

A light yellow crystal of the title compound with approximate dimensions of 0.50 × 0.42 × 0.41 mm was selected for data collection on an Bruker SMART 1997 CCD diffractometer with a graphite-monochromatized MoKα (λ = 0.71073 Å) radiation. A total of 2776 reflections were collected in the range of 2.03º < θ < 25.01º by using an ω-2θ scan mode at 293(2) K.of which 1849 reflections were independent with Rint = 0.0148 and 1284 observed reflections with I > 2σ(I) were used in the succeeding refinements. The structure was solved by direct methods and expanded using Fourier difference techniques with SHELXTL-97 program package [8]. The non-hydrogen atoms were refined anisotropically by full-matrix least-squares calculations on F^2^. Details of the crystal parameters, data collection and refinement are summarized in Table 1.

Supplementary crystallographic data have been deposited with the Cambridge Crystallographic Data Centre as CCDC No. 783257. Copies of this information may be obtained free of charge from The Director, CCDC, 12 Union Road, Cambridge CB2 1EZ, UK (Fax: +44 1223 336033; Email: deposit@ccdc.cam.ac.uk or www: http://www.ccdc.cam.ac.uk).

### 3.5. Biological activity

#### 3.5.1. Cell culture

Growth inhibitory activity of the title compound was bio-evaluated *in vitro* on four different cell lines: BEL-7402 (human hepatocellular carcinoma BEL-7402 cells), K562 (human leukemia cell line K562), SPC-A-1 (human lung adenocarcinoma SPC-A-1 cells), SGC-7409 (human gastric carcinoma cell SGC-7901).

#### 3.5.2. Cytotoxicity assay

Chemosensitivity of these cells to the title compound was determined by MTT microculture tetrazolium assay, as described by Mossmann [9]. Briefly, cells were harvested at exponential growth phase and were seeded in flat bottom 96-well plates. The cell volume in each well was 180 mL, BEL-7402 contained 7 × 10^4^ cells per well; SGC-7409 contained 4 × 10^4^ cells per well; SPCA-1 contained 2 × 10^4^ cells per well; K562 contained 10^4^ cells per well. The plates were incubated overnight in a 5% CO_2_ incubator at 37 ºC. The compounds were then added to each well at various concentrations using a constant volume of 20 mL, in triplicate, and maintaining a total well volume of 200 mL. After 48 h incubation at 37 ºC in 5% CO_2_ concentration, 25 mL of MTT (5 mg/mL in PBS) was added to each well and again incubated at 37 ºC for 4 h. After removing the medium carefully by aspiration, 150 μL of DMSO was added to each well and the formazan dye crystals were dissolved by shaking gently for 15 min. The plates were then read at 570nm wavelength in a microplate reader. IC_50_ values of the compounds in different cell lines were determined, based on dose response curve. The results are summarised in Table 4.

## 4. Conclusions

We reported the iso9lation of marcanine A from the genus *Polyalthia* for the first time. Its chemical structure was elucidated on the basis of physicochemical properties and spectral data including IR, ^1^H-NMR,^13^C-NMR and HMBC. The crystal structure has been determined by X-ray diffraction analysis. It crystallizes in the triclinic system, space group P1 with a = 5.2140(5)Å, b = 10.1871(11)Å, c = 11.0709(13)Å, α = 110.452(2)º, β = 103.376(2)°, γ =90.1870(10)°, V =533.74(10)Å^3^, Z = 2, Dx = 1.489 g/cm^3^. Beside the four kinds human tumor cells (BEL-7402, K562, SPCA-1 and SGC-7409) tested in this work, we can also learn from the literature [4] that it has effect on five other kinds cells, ncluding A-549, HT-29, MCF7, RPMI and U251. The title compound is an interesting natural product with some inhibitory activities towards many human tumor cells.
